# Evaluation of secondary surgery to enlarge the peeling of the internal limiting membrane following the failed surgery of idiopathic macular holes

**DOI:** 10.3892/etm.2014.1477

**Published:** 2014-01-07

**Authors:** XIN CHE, FANGLIN HE, LINNA LU, DONGQING ZHU, XIAOFANG XU, XIN SONG, XIANQUN FAN, ZHILIANG WANG

**Affiliations:** 1 Department of Ophthamology, Ninth People’s Hospital Affliated with Shanghai Jiaotong University, Shanghai 200011, P.R. China; 2 Key Laboratory of Ophthamology, Ninth People’s Hospital Affliated with Shanghai Jiaotong University, Shanghai 200011, P.R. China

**Keywords:** idiopathic macular holes, internal limiting membrane peeling enlargement surgery, pars plana vitrectomy

## Abstract

The aim of the present study was to evaluate the clinical results of pars plana vitrectomy (PPV) combined with the surgical enlargement of internal limiting membrane (ILM) peeling in patients who had previously undergone failed idiopathic macular hole (IMH) surgery. In the study, 134 eyes from 130 IMH patients who had received PPV combined with ILM peeling surgery (2 disk diameters) were analyzed. Within this cohort, 14 eyes had IMHs that were not closed, of which 13 eyes underwent a second surgery involving enlargement of the ILM peeling. The extent of the ILM peeling was increased to the vascular arcades of the posterior fundus in the secondary surgery. Of the 13 eyes that underwent secondary surgery, five were in stage III and nine were in stage IV. The second surgery successfully achieved IMH closure in 61.5% (8/13) of the eyes. The IMH was completely closed following surgery and the logMAR vision increased from 0.98 to 0.84 (P=0.013) in the 8 successfully treated cases. The surgical enlargement of ILM peeling closed the IMHs and improved vision in the majority of patients. In addition, the procedures were safe. Therefore, the results of the present study indicate that enlargement of ILM peeling may be an effective therapy for patients who have previously undergone the failed surgical correction of an IMH.

## Introduction

Idiopathic macular holes (IMHs), which lack a precise ocular pathogenesis that is likely to indicate a primary disease of the fundus, have been identified as a cause of visual loss in the elderly. The macula lutea is the most important area for central vision. A hallmark inciting event for IMH formation is considered to be focal shrinkage of the vitreous cortex and traction in the foveal area. Subsequently, central visual acuity usually deteriorates. Kelly and Wendel were the first to report improved results for vitreous surgery of an IMH in 1991 (1). Since then, vitrectomy for IMHs has rapidly become a commonly performed procedure worldwide. Currently, the majority of scholars consider that a combined application of vitrectomy with internal limiting membrane (ILM) peeling is able to improve anatomical closure and the recovery of vision. However, a series of studies have shown that combined surgery results in IMH closure in 90% of eyes, which indicates that 10% of eyes failed to achieve IMH closure (2–6). The development of methods to prevent failure and promote anatomical closure is currently a challenging issue in clinical treatment (7,8). Our research group has developed a surgical procedure for the enlargement of ILM peeling combined with vitrectomy following a previous failed correction of IMH. The present study investigates the efficacy of this secondary surgery for macular hole closure.

## Materials and methods

### Clinical data

Between January 2009 and June 2011, patients who received a pars plana vitrectomy (PPV) combined with ILM peeling surgery were studied in the Department of Ophthalmology of the Ninth People’s Hospital affiliated with Shanghai Jiaotong University (Shanghai, China). All patients provided informed consent prior to surgery. The present study was approved by the Ethics Committee of the Ninth People’s Hospital affiliated with Shanghai Jiaotong University. A slit lamp with a handheld or fixed lens and optical coherence tomography (OCT; Cirrus™ HD-OCT 4000; Carl Zeiss Meditec, Inc., Jena, Germany) were used to examine the periphery and the center of the retina. All patients were diagnosed as having an IMH. The exclusion criteria included myopia of >6 diopters, ocular trauma and other systematic or ocular issues associated with IMHs. The series included 134 eyes of 130 patients, of which 42 patients were male and 88 were female with 42 and 92 eyes being treated, respectively. The age range was 46–81 years, with a mean of 63.75±7.45 years. Patient clinical course ranged between 1 and 36 months, with a mean of 4.4±5.3 months. The eyes were divided into stages according to the Gass categories (9). There were 23 eyes (17.1%) in stage II, 47 eyes (35.1%) in stage III and 64 eyes (47.8%) in stage IV. Retinal defects ranged between 200 and 800 μm, in diameter with a mean of 457±129 μm. In total, 134 eyes underwent the standard three-port full PPV using a 23-gauge incision, combined with ILM peeling. This was followed by phacoemulsification and artificial lens implantation. Following the application of 0.025% indocyanine green (ICG) dye (Dandong Medical and Pharmaceutical Ltd. Liability Corporation, Dandong, China) during surgery, ILM of 2 disk diameters (DD) around the hole was peeled off. Among these eyes, 14 retinal defects were not closed, of which 11 IMHs remained open at week 1. In addition, three reopened 3 months postoperatively. Among the 14 patients, one declined secondary surgery and the remaining 13 cases agreed to have a secondary vitrectomy with surgical enlargement of ILM peeling.

The 13 eyes that underwent secondary ILM peeling surgery (four male and nice female) each had an artificial lens. Patient age ranged between 43 and 79 years, with a mean of 64.65±6.18 years and the clinical course ranged between 6 and 30 months with an mean of 13.4±5.4 months. IMHs ranged between 450 and 800 μm in diameter and the mean was 584±89 μm. The 13 eyes comprised five in stage III and nine in stage IV.

### Surgery methods

Primary and secondary surgeries were performed by one surgeon. All surgeries were performed under local anesthesia. The primary surgeries were a standard three-port full PPV using 23-gauge incisions, phacoemulsification and artificial lens implantation in phakic eyes. The posterior cortical vitreous was removed using triamcinolone acetonide to aid visualization. ICG (0.1 ml, 0.025%) was applied to stain the macula lutea for 15 sec, and was then removed by suctioning. The extent of the ILM peeling was 2–4 DD and 14% C_3_F_8_ gas was introduced into the vitreous cavity during surgery. Patients were instructed to maintain a strict face-down position for 3 h/day for 7 days postoperatively. Secondary surgeries to enlarge the peeling of the ILM were performed using a standard three-port 23-gauge incision following local anesthesia; 0.1 ml ICG (0.025%) was injected into the vitreous cavity and later removed by suctioning. Following this procedure, the ILM edge remaining from the previous surgery was viewable. This edge was engaged with retinal forceps tangentially to the retinal surface and the ILM was torn annularly to the vascular arcades of the posterior retina. C_3_F_8_ (14%) was then administered to the vitreous cavity. Patients were instructed to remain face-down for 3 h/day for 7 days.

### Clinical observations

Eyes were examined at week 1, months 1, 3 and 6 and 1 year following surgery. Snellen chart assessments, as well as slit lamp examinations combined with a three-mirror contact lens and a 120-diopter lens were performed to examine vision, the intraocular anterior segment and the ocular fundus. OCT was used to observe the macular configuration and determine if there were any complications. In addition, patients were closely observed for 6 months to check for the development of a retinal hole. An anatomical success was defined as an OCT examination showing the complete disappearance of the hole and the recovery of continuity in the neural epithelium. If the hole was not completely closed or the retinal neurosensory layer lacked integrity, the surgery was determined to have failed, even if the neurosensory layer was anatomically closed and the hole had become smaller.

### Statistical analysis

Visual acuity scores from pre- and postsurgery were transformed to logMAR and subsequently the vision of index and hand motion corresponded to 2.0 and 3.0, respectively. A t-test was performed to compare variables among various groups, including age, duration of the disease, size of the IMH and the time of follow-up between the first surgery with a vitrectomy and ILM peeling surgery and the second with a vitrectomy and surgery to enlarge ILM peeling. The Gass stages were analyzed with a χ^2^ test. P<0.05 was considered to indicate a statistically significant difference. The results presented in this study are expressed as the mean ± standard deviation (SD).

## Results

### Postsurgical ocular conditions

Table I shows the characteristics of gender, age, follow-up time, Gass stage, size of the IMH and pre and postsurgical acuity of patients at the primary surgery, involving PPV combined with ILM peeling, and the secondary surgery to enlarge the ILM peeling. There was no significant difference in gender, age and follow-up time between the primary and secondary surgeries. The duration of the clinical course in the secondary surgery group was 13.3±4.0 months, which was significantly different from the duration course of 4.5±5.0 months for the primary surgery group. In addition, the IMHs in the secondary surgery patients were larger, with a mean size of 584±89 μm, compared with those in the patients treated with the first surgery, which had a mean size of 457±129 μm.

**Figure 1:**
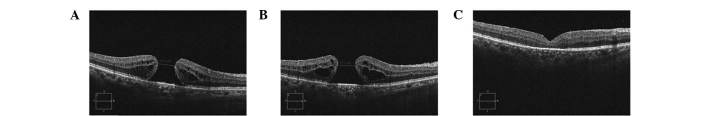
Serial OCT scans showing a patient with a stage IV macular hole. (A) Preoperative OCT line scan demonstrates a full-thickness macular hole. (B) 2 DD of the ILM centered on the macular hole was peeled in the first PPV and the macular hole did not close. (C) ILM peeling for the secondary surgery was enlarged to the vascular arcades of the posterior fundus and the macular hole was closed. OCT, optical coherence tomography; DD, disc diameter; ILM internal limiting membrane; PPV, pars plana vitrectomy.

**Figure 2:**
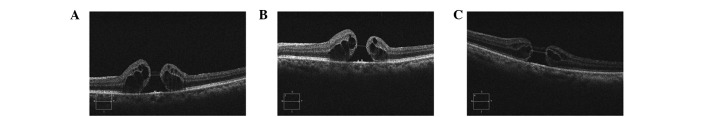
Serial OCT scans showing a patient with a stage III macular hole. (A) Preoperative OCT line scan demonstrates a full-thickness macular hole. (B) 2 DD of the ILM centered on the macular hole was peeled in the first PPV and the macular hole was not closed. (C) ILM peeling for the secondary surgery was enlarged to the vascular arcades of the posterior fundus and the macular hole was not closed. Cystic changes remained unresolved. OCT, optical coherence tomography; DD, disc diameter; ILM internal limiting membrane; PPV, pars plana vitrectomy.

**Figure 3:**
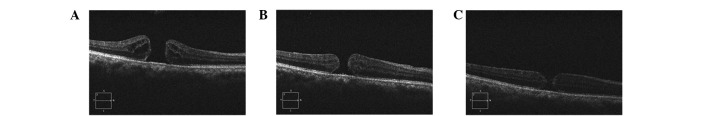
Serial OCT scans showing a patient with a stage IV macular hole. (A) Preoperative OCT line scan demonstrates a full-thickness macular hole with cystic changes. (B) 2 DD of the ILM centered on the macular hole was peeled in the first PPV and the macular hole was not closed. (C) ILM peeling for the secondary surgery was enlarged to the vascular arcades of the posterior fundus and the macular hole did not close but became flat with the cystic changes resolved. OCT, optical coherence tomography; DD, disc diameter; ILM internal limiting membrane; PPV, pars plana vitrectomy.

For all the primary surgery patients, the rate of anatomical closure of the IMH was 89.6% (120/134). In addition, IMH closure was achieved in 100% (23/23), 89.4% (42/47) and 85.9% (55/64) of the patients in stages II, III and IV, respectively. There were 14 eyes that did not heal in 14 patients, of which five were in stage III and nine were in stage IV. Excluding the one patient in stage IV who declined the secondary surgery, the rate of closure was 61.5% (8/13) in the group of 13 patients that underwent the secondary surgery to enlarge the ILM peeling (Fig. 1). In this group, the success rate was 80% (4/5) in stage III and 50% (4/8) in stage IV. There were five eyes in five patients in which the IMHs did not close completely (Fig. 2), of which three had similar morphology and size postoperatively. The remaining two eyes had a retinal neurosensory layer attached to the retinal pigment epithelium (RPE), but a smaller hole (Fig. 3).

### Postsurgical visual acuity

The mean logMAR visual acuity following the primary surgery increased from 0.94±0.19 to 0.61±0.28 (P<0.001), which was considered to be statistically significant. Following the secondary surgery, the mean visual acuity increased from 1.03±0.18 to 0.92±0.16 (P=0.021), a statistically significant difference. Symptoms of metamorphopsia disappeared or were alleviated in the eight eyes with successful anatomical closure and visual acuity was 0.98 prior to surgery and 0.84 six months following surgery, a statistically significant difference (P=0.013). The five unclosed eyes showed slightly improved vision 6 months following the surgical enlargement of ILM peeling; the mean visual acuity of 1.026 was improved to 1.010. This difference was not found to be significantly different (P=0.186).

### Complications following surgery

The secondary surgeries to enlarge ILM peeling were completed in the 13 eyes, during which there was bleeding in eight eyes. However, the bleeding stopped spontaneously. Retinal tears or active hemorrhage were not present during the course of the surgeries. Patients were followed-up for 6–28 months, with a mean follow-up duration of 13.5±1.3 months. During the follow-up period, no complications, including bleeding or retinal tears, were observed.

## Discussion

ILM peeling surgery began in 1992 with the hypothesis that it may be used to close IMHs and prevent them from reopening (10). The ILM is a layer of transparent film, located between the retina and the vitreous humor and has been hypothesized to be composed of a basement membrane of Müller cells (11). The ILM is thick in the area of the macula, but extremely thin in the central fovea of the macula. It is closely attached to the vitreous cortex. Pathologically, the ILM supports the proliferation of RPE cells and fibroblasts. ILM peeling surgery removes the ILM and tissue that is overexpressed and loosens the traction in a tangential direction around the hole, which promotes the healing of the IMH (4). This also clears support for the proliferation of RPE and fibroblasts, avoiding the emergence of the epiretinal membrane, which may prevent the recurrence of an IMH (5). In addition, the trauma produced by ILM peeling may stimulate the proliferation of gliocytes, including Müller cells, in favor of closing the IMH (6). However, other methods to promote the closure of IMHs in patients who have undergone failed surgeries must be identified to further improve vision.

In 2008, Valldeperas and Wong performed secondary surgery with autonomous plasma and gas as a filler in patients with failed closure of an IMH. Anatomical closure was achieved in 76% (40/52) of the patients (7). The difference between the authors’ findings and those of the present study may be due to technique. In the 2008 study, ILM peeling was rarely carried out in the initial and secondary surgeries. In 2011, D’Souza *et al* reported the rate of anatomical closure as 46.7% (14/30) following secondary surgery with PPV and ILM peeling in cases where the first surgery had failed (8). The study by D’Souza *et al* described the surgeries for a number of cases that were performed by three surgeons, but provided no detailed description of the size of the area that was peeled in the primary and secondary surgeries. The success rate of the surgeries to enlarge ILM peeling performed in the present study, which was 61.5% (8/13) for IMH closure, markedly exceeded the success rate in the study by D’Souza *et al*. This difference may be associated with the extent of the enlargement in the peeling surgery; in the current study, the peeling extended to the vascular arcades of the posterior fundus. It has been hypothesized that enlargement of the peeling by surgery may loosen the tangentially directed traction coming from the peripheral retina, which acts on the retina around the IMH. In addition, the absence of traction for the posterior fundus is beneficial for the neuroepithelium attached to the RPE. Also, the trauma produced by peeling the ILM promotes the proliferation of gliacytes, including Müller cells, which promotes the healing of the IMH.

It was observed that visual acuity markedly increased following the secondary closure; however, the acuity was considerably less than that prior to IMH closure following the primary surgery, which is consistent with a previous study by Christensen *et al* (12). Restoration of the anatomy and function of the macular neuroepithelium is likely to result in the gradual improvement of visual acuity between 6 and 12 months following surgery (13–15). This indicates that the primary focus should be on closing the hole in IMH surgeries, rather than seeking a 50:50 chance of closing the hole without ILM peeling in the search for a slightly improved functional outcome. In addition, certain studies have shown that the removal of the ILM may have a possible risk of mechanical retinal damage and toxic damage due to the use of dyes or illumination (16–20). However, ILM removal was not identified to have an effect on visual acuity in IMH surgery (21).

Following the first ILM peeling surgery, the unhealed eyes were in stage III or IV, their course of disease was >12 months and the size of the IMH was >450 μm. These observations were significantly different to those of the healed eyes following the primary surgery. This indicates that the closure of the IMH is closely associated with the stage and duration of the disease and the size of the IMH. These results are consistent with the study by Kumagai *et al* in which the rate of macular hole closure in Asian individuals was inversely associated with the duration of disease when the duration was >6 months and the size of the IMH was >400 μm (22). It remains controversial whether there is a correlation between the success of IMH closure and the course of the disease with the size of IMH (23,24). Further studies are required to determine if the duration and size of the IMH affects the closing of IMHs in Caucasians or Asians. The method in the present study promoted the closure of IMHs in two-thirds of the unhealed IMHs and no complications were observed during the surgeries. Therefore, it remains questionable whether there is a need to increase the extent of the peel from 2 DD to the size of the vascular arcades of the posterior fundus in stage III or IV patients with a clinical course of >12 months and an IMH size of >450 μm.

In conclusion, in cases where an IMH fails to close following vitrectomy combined with ILM peeling surgery, a secondary surgery to extend the peeling of the ILM to the vascular arcades of the posterior fundus is recommended. The secondary surgery effectively promotes the closure of the IMH and also anatomical reset. The secondary surgery is relatively safe. The results obtained indicate that for the patients with a clinical course of >12 months and a macular hole size >450 μm in stages III or IV, it may be favorable to increase the extent of the peel from 2 DD to the size of the vascular arcades during the primary surgery. Due to the current study being a retrospective study with only a small sample size, a prospective study is currently being conducted to evaluate the clinical results and the safety of primary PPV combined with enlargement of ILM peeling for patients with long-term, large IMHs in stages III or IV.

## Figures and Tables

**Table I:** Baseline characteristics and postoperative functional outcomes in eyes following the first PPV surgery and the second surgery to enlarge ILM peeling following first surgery failure.

Characteristics	First surgery	Second surgery	t-value	P-value
Gender				
Male	42	4		
Female	88	9	0.013	0.91
Age, years	64.2±7.1	64.6±6.1	1.54	0.14
Duration, months	4.5±5.0	13.3±4.0	6.13	<0.001
Gass stage				
II	23	0		
III	47	5		
IV	64	9	1.64	0.10
Macular hole size, μm	443.0±122.6	588.5±108.3	4.12	<0.001
Follow-up, months	12.3±0.88	13.5±5.8	0.70	0.497
LogMAR, presurgery	0.94±0.19	1.03±0.18	1.60	0.08
LogMAR, postsurgery	0.61±0.28	0.92±0.16	6.16	<0.001

PPV, pars plana vitrectomy; ILM, internal limiting membrane.
